# Epidemic trend of *Salmonella* from swines and broilers in China from 2014 to 2023 and genetic evolution analysis of ESBLs-producing strains

**DOI:** 10.3389/fmicb.2025.1510751

**Published:** 2025-02-14

**Authors:** Yaopeng Liu, Lin Wang, Juan Wang, Mingzhe Lu, Na Liu, Jianmei Zhao, Fangyuan Hu, Keguang Han, Junhui Liu, Junwei Wang, Zhina Qu

**Affiliations:** ^1^China Animal Health and Epidemiology Center, Qingdao, China; ^2^College of Veterinary Medicine, Shanxi Agricultural University, Taigu, China

**Keywords:** *Salmonella*, swine, broiler, antimicrobial resistance, ESBLs

## Abstract

**Introduction:**

In recent years, the epidemic trend and antimicrobial resistance of *Salmonella* from swines and broilers, especially the extended-spectrum β-lactamase (ESBLs)-producing *Salmonella*, pose a serious threat to human and animal health.

**Methods:**

In this study, we employed serotype identification, drug sensitivity testing, detection of ESBL-producing strains, and whole genome sequencing to analyze the epidemiological trends and drug resistance of *Salmonella* isolates from swines and broilers, as well as the genetic evolutionary relationships of ESBL-producing strains in China from 2014 to 2023.

**Results:**

The results showed that the most prevalent serotypes of *Salmonella* from swines and broilers in China in recent 10 years were *S. Typhimurium* (133/381, 34.91%) and *S. Enteritidis* (156/416, 37.50%), respectively. Overall, 80.58% strains from swines and 70.67% strains from broilers were multidrug resistant. The multidrug resistance rate (MDR) showed a downward trend. The types of drugs exhibiting an increasing trend in resistance rates among *Salmonella* from broilers (7) were significantly greater than those from swines (2). The detection rates of ESBLs-producing *Salmonella* from swines and broilers were 9.45 and 29.58%, respectively, with the former showing a downward trend and the latter showing an upward trend. The drug resistance phenotype of *Salmonella* produced in ESBLs from swines and broilers is consistent with the results of the resistance genes carried. Whole genome sequencing analysis revealed that 36 swine-derived ESBL-producing *Salmonella* strains contained 6 ST-types and 13 cgST-types, among which ST34 and ST17 were dominant ST-types; a total of 35 resistance genes across 11 classes, *blaCTX-M-14*, *blaTEM-1B*, and *blaCTX-M-65* were the predominant subtypes of β-lactam resistance genes. 126 broiler-derived ESBL-producing *Salmonella* strains included 19 ST-types and 37 cgST-types, with ST17 and ST198 as the dominant ST-types; a total of 52 resistance genes belonging to 12 classes, *blaCTX-M-55*, *blaCTX-M-65*, *blaTEM-1B*, and *blaOXA-1* identified as the major subtypes of β-lactam resistance genes.

**Discussion:**

This suggests that we should thoroughly implement management policies aimed at reducing the use of veterinary antimicrobials. Additionally, we should enhance research on traceability technology and the abatement of resistance genes, thereby providing support for the effective prevention and control of the spread of *Salmonella* and its drug resistance.

## 1 Introduction

*Salmonella* is a zoonotic intestinal pathogen, which can cause a wide range of diseases in both humans and animals. The World Health Organization (WHO) classifies it as a foodborne pathogen of serious and moderate harm. Up to now, more than 2,600 serotypes of *Salmonella* have been identified, many of which are potentially pathogenic and are widely distributed in nature (Whiley and Ross, 2015). In recent years, the widespread use of antibiotics has led to a year-on-year increase in the resistance rate of *Salmonella* to these drugs. Van Boeckel et al. (2019) reported that between 2000 and 2018, the proportion of *Salmonella* from broilers that exhibited resistance to more than 50% of antimicrobials rose from 15 to 41%. Conversely, the proportion of swine-origin *Salmonella* with similar resistance increased from 13 to 34%. Some antibiotics that were once considered effective, such as doxycycline, gentamicin, and mucomycin, have gradually lost their efficacy. The phenomenon of multidrug resistance has become increasingly serious, which is mainly due to the unreasonable use of antibiotics in both humans and animals (Aslam et al., 2021; Tang et al., 2023). This issue is further exacerbated by the emergence of extended-spectrum beta-lactamase (ESBL)-producing *Salmonella*. In 2000, Mulvey et al. (2003) detected only one ESBL-producing strain among over 1,000 *Salmonella* isolates. In 2010, Wu et al. (2015) reported that the detection rate of ESBL-producing *Salmonella* in retail raw broiler meat was 8.5%. ESBL-producing strains possess the ability to hydrolyze third- and fourth-generation cephalosporins, making commonly used antibiotics ineffective and significantly exacerbating the challenge of antibiotic resistance in *Salmonella*. Antimicrobial resistance (AMR) has been widely described as a “silent epidemic” and is now recognized by the One Health approach as a major risk factor for global health. In 2019, approximately 4.95 million deaths globally were related to AMR bacteria, with projections indicating that this number could rise to 10 million by 2050 (Murray et al., 2022).

Whole genome sequencing (WGS) is a crucial tool for monitoring bacterial infectious diseases and elucidating the transmission routes of pathogens. It plays a significant role in the detection, prediction, research, and monitoring of AMR, as well as in the prevention and control of these infections. It is commonly employed to trace the origin of pathogenic bacteria. WGS facilitates serotyping, multilocus sequence typing (MLST), and core genome MLST (cgMLST) typing, as well as querying antimicrobial resistance genes with high resolution and accuracy (Inns et al., 2017). This technology has been successfully utilized for monitoring and tracing the prevalence of *Salmonella*. Over the past decade, China’s animal husbandry industry has experienced rapid development and remarkable expansion, which has enhanced the utilization and regulation of vaccines and veterinary drugs. However, the unreasonable use of veterinary drugs have also been reported, leading to the emergence of bacterial drug resistance. Consequently, this study integrates China’s breeding production and geographical distribution by selecting 14 representative provinces. It employs swine- and broiler-derived *Salmonella* isolates from swines and broilers from 2014 to 2023 were used as the research subjects, and the serotype identification, drug sensitivity testing, and ESBLs production were carried out. The study includes strain detection and whole gene sequencing to analyze the drug resistance and epidemic trends of *Salmonella* from swines and broilers in China in recent ten years, and the genetic evolution of ESBL-producing strains. The purpose of this study is to clarify the epidemic trends of *Salmonella* in this context, promote rational drug use and solve the drug resistance problem in animal breeding, thus providing support for the effective prevention and control of drug-resistant bacteria.

## 2 Materials and methods

### 2.1 *Salmonella* isolates

**FIGURE 1:**
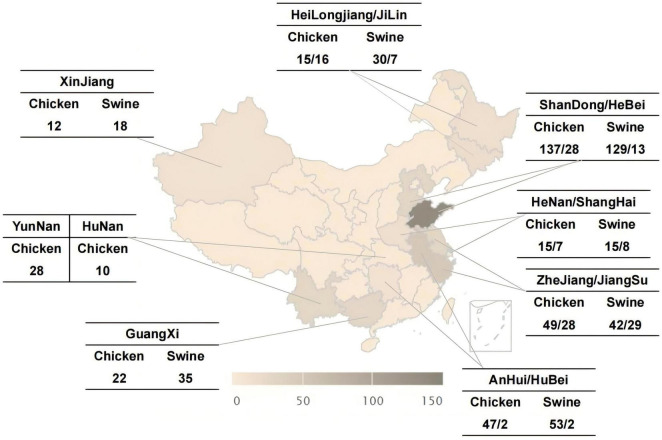
Distribution of *Salmonella* isolates from swines and broilers in 14 provinces in China, 2014–2023.

A total of 381 swine-origin *Salmonella* strains and 416 broiler-origin *Salmonella* strains were isolated from anal swab samples of swine and cloacal swab samples of broilers collected across 14 provinces, namely Heilongjiang, Jilin, Shandong, Hebei, Henan, Shanghai, Zhejiang, Jiangsu, Anhui, Guangxi, Hubei, Xinjiang, Yunnan, and Hunan, during the period from 2014 to 2023. The distribution of strains was shown in Figure 1. The standard strains used in this study were *Salmonella* ATCC14028, *Escherichia coli* ATCC25922, and *Klebsiella pneumoniae* ATCC700603. All strains were supplied by the Pathogenic Microorganisms Surveillance Unit of the Chinese Animal Health And Epidemiology Centre.

### 2.2 Identification of *Salmonella* serotypes

The quality of the extracted DNA was assessed by measuring the absorbance at 260 nm and 280 nm, and calculating the A260/A280 ratio in three replicates using a spectrophotometer. This was accomplished through the boiling method established by Rathnayaka (2011) for the extraction of DNA from the *Salmonella* standard strain ATCC 14028 and isolates used as templates for the PCR reaction. Serotyping was conducted according to the Kauffman-White Diagnostic Antigen Table for *Salmonella* spp., referencing the PCR method developed by Zhao et al. (2018).

### 2.3 Antimicrobial susceptibility testing

In accordance with the standards set forth by the American Clinical and Laboratory Standards Institute (Clinical Laboratory Standards Institute (CLSI), 2020), the minimal inhibitory concentration (MIC) of the *Salmonella* isolates in step 3.1 was determined using the microbroth dilution method for 14 different antibacterial agents across 10 categories. The 14 antimicrobial drug classes and their corresponding resistance fold-point concentrations are as follows: Ampicillin (AM, 0.25-512), Amoxicillin-clavulanic acid (A/C, 0.25/0.12-512/256), Tetracycline (TE, 0.25-512), Gentamicin (GM, 0.25-512), Florfenicol (FFC, 0.25-512), Spectinomycin (SPT, 0.25-512), Sulfisoxazole (SF, 0.25-512), Sulfamethoxazole and Trimethoprim (SXT, 0.06/1.2-32/608), Ceftiofur (CEF, 0.12-256), Ceftazidime (CAZ, 0.12-256), Enrofloxacin (ENR, 0.015-32), Ofloxacin (OFL, 0.03-64), Colistin (CL, 0.12-256), and Meropenem (MEM, 0.008-16). *Salmonella* isolates were inoculated onto Tryptic Soya Peptone Agar Medium (TSA), and after incubation for 18 h at 37°C, 2–3 colonies were selected and transferred into 2–3 mL of sterilized saline. In the saline, a 0.5 McFarland turbidity standard was utilized to adjust the concentration of the bacterial solution to 1.5 × 10^8^ cfu/mL. Subsequently, 60 μL of this bacterial solution was mixed with 12 mL of the drug sensitivity culture solution for dilution. A 100 μL aliquot of the diluted solution was then added to a 96-well drug sensitivity plate, which was incubated at a constant temperature of 35°C for 16–20 h for result assessment. *E. coli* ATCC 25922 was employed as the quality control organism. The results were considered valid if the quality control strain fell within the established quality control range, with bacterial growth observed in the positive control and no growth in the negative control. Multi-resistance was defined as simultaneous resistance to three or more classes of antimicrobials.

### 2.4 Identification of ESBL producing strains

In accordance with the standards set forth by the American Committee for Clinical and Laboratory Standards (Clinical Laboratory Standards Institute (CLSI), 2020), *Salmonella* isolates were assessed for the production of extended-spectrum beta-lactamases (ESBLs) using the paper diffusion method, specifically the Kirby-Bauer (K-B) method. A total of 3–4 colonies were selected and evenly spread on Mueller-Hinton agar (MHA) plates. Sterile forceps were utilized to apply cefotaxime (CTX), ceftriaxone (CRO), ceftazidime (CAZ), and aminotrimoxazole (AZT) onto the surface of each plate. The aforementioned antibiotics, including Ceftazidime/Clavulanic Acid (CAZ/CA) and Cefotaxime/Clavulanic Acid (CTX/CA) combinations, were uniformly affixed to the plates using sterile tweezers. Following incubation at 37°C for 16–18 h, the diameters of the inhibition zones were measured. Enterobacteriaceae ATCC25922 served as the negative quality control, while Klebsiella pneumoniae ATCC700603 was used as the positive quality control. Interpretation of results: If the quality control strains fell within the acceptable range and the diameter of the inhibition zones of the tested strains met any of the following criteria: CAZ ≤ 22 mm, CTX ≤ 27 mm, CRO ≤ 25 mm, AZT ≤ 27 mm, it was suggested that the strain was a potential ESBL-producing *Salmonella*. Additionally, if the diameter of the inhibition zone for any group of strains treated with CA sensitivity tablets was ≥ 5 mm greater than that of the strains without CA sensitivity tablets, the strain was confirmed as an ESBL-producing *Salmonella*.

### 2.5 Whole genome sequences analysis of *Salmonella* strains

The 3.4 ESBL-producing strains were purified and cultured, and the bacteria were subsequently sent to (BGI, Wuhan, China) for whole genome sequencing and assembly. Library preparation: DNA extraction was performed on the genome, and the whole genome DNA was randomly fragmented to an average size of 200–400 bp. After purification with the magnetic beads, the library was qualified by the fluorometer and the length of library was assessed by the 2% agarose gel electrophoresis. The qualified libraries were sequenced on the MGISEQ-2000 sequencing platform. The sequencing quality was ensured by filtering the low-quality data from MGISEQ, assembling the samples on Clean Date based on the valid data available on the sequencing platform, and evaluating the valid assembly data by GC-Depth.

The BacWGSTdb website^1^ was utilized to query the isolates for ST-type comparisons and resistance genes. Additionally, the CGE website^2^ was employed to predict the serotypes and cgMLST types of the isolates using the SeqSero and cgMLSTFinder modules. Bionumerics software was used to construct minimum spanning trees of MLST and cgMLST of the *Salmonella* isolates. Sequence comparison and SNP identification were performed on the isolates to construct core genome evolutionary trees, and the cluster data were imported into Chiplot with Newick for further analysis.

### 2.6 Data analysis

The distribution of *Salmonella* isolates from 2014 to 2023 was produced using the R4.3.2 ggplot2 package. Statistical AMR for each drug tested (95% confidence interval) using Minitab Statistical Software; summary analysis of *Salmonella* multi-drug resistance using Graphpad Prism 8.0. Differences were analyzed for significance by chi-square test using SPSS Statistics 23 software (*P* < 0.01 high significance, *P* < 0.05 significant, *P* > 0.05 insignificant difference).

## 3 Results

### 3.1 Identification of *Salmonella* serotypes

From 2014 to 2023, the serotype identification results of 381 strains of *Salmonella* from swines and 416 strains of *Salmonella* from broilers were shown in Tables 1, 2. A total of 32 serotypes were identified from 381 strains of *Salmonella* from swines. The dominant serotypes were determined, and the distribution of *Salmonella* serotypes across different years was analyzed. The most prevalent serotype was *S. Typhimurium* (133/381, 34.90%), followed by *S. Risen* (62/381, 16.27%) and *S. Derby* (61/381, 16.01%). The dominant serotype was *S. Typhimurium* in all the years except in 2014 for *S. Farsta* (60.00%), in 2015 for *S. Indiana* (25.00%) and in 2019 for *S. Derby* (36.36%). A total of 38 serotypes were identified in the 416 broiler-origin *Salmonella* strains, with the dominant serotype being *S. Enteritidis* (156/416, 37.50%), followed by *S. Typhimurium* (35/416, 8.41%) and *S. Indiana* (34/416, 8.17%). Except for *S. Kentucky* in 2017 (31.58%) and 2022 (23.81%) and *S. Infantis* in 2023 (24.07%), the dominant serotype was *S. Enteritidis* in all years.

**TABLE 1:** Serotype identification of 381 strains of *Salmonella* from swines in 2014–2023.

2014 *n* = 5	%	2015 *n* = 16	%	2016 *n* = 25	%	2017 *n* = 31	%	2018 *n* = 26	%	2019 *n* = 66	%	2020 *n* = 78	%	2021 *n* = 45	%	2022 *n* = 43	%	2023 *n* = 46	%
*S. Farsta*	60	*S. Indiana*	25	*S. Typhi-* *murium*	72	*S. Typhi-* *murium*	48.38	*S. Typhi-* *murium*	57.69	*S. Derby*	36.36	*S. Typhi-* *murium*	37.18	*S. Typhi-* *murium*	24.44	*S. Typhi-* *murium*	39.53	*S. Typhi-* *murium*	30.43
*S. Potsdam*	40	*S. Rissen*	18.75	*S. Derby*	16	*S. Enteritidis*	35.48	*S. Derby*	23.07	*S. Typhi-* *murium*	21.21	*S. Rissen*	25.64	*S. Rissen*	31.11	*S. London*	20.93	*S. Derby*	39.13
		*S. Derby*	12.5	*S. Bury*	8	*S. Indiana*	9.67	*S. Infantis*	7.69	*S. Rissen*	10.6	*S. California*	17.95	*S. London*	15.55	*S. Rissen*	20.93	*S. Rissen*	17.39
		*S. Kunduchi*	12.5	*S. Rissen*	4	*S. Goldcoast*	3.22	*S. Welte-* *vreden*	3.84	*S. Tsevie*	4.54	*S. Derby*	5.13	*S. Schwarzen-* *grund*	4.44	*S. Derby*	6.97	*S. Braen-* *derup*	8.69
		*S. Kubacha*	6.25			*S. Welte-* *vreden*	3.22	Others	7.71	*S. Augusten-* *borg*	3.03	*S. Goettingen*	2.56	*S. Stanley*	4.44	*S. Goldcoast*	2.32	Others	4.36
		*S. Gloucester*	6.25							*S. Berta*	1.51	*S. Newport*	2.56	*S. California*	2.22	*S. Reading*	2.32		
		*S. Lagos*	6.25							*S. Drogana*	1.51	Others	8.97	*S. Newport*	2.22	Others	7		
		*S. Essen*	6.25							*S. Enteritidis*	1.51			*S. Tumodi*	2.22				
		Others	6.25							*S. Gloucester*	1.51			Others	13.33				
										*S. Indiana*	1.51								
										*S. Infantis*	1.51								
										*S. Lagos*	1.51								
										*S. London*	1.51								
										*S. Concord*	1.51								
										*S. California*	1.51								
										*S. Seremban*	1.51								
										*S. Cuckmere*	1.51								
										Others	6.14								

**TABLE 2:** Identification of *Salmonella* serotypes from broilers of 416 strains, 2014–2023.

2014 *n* = 45	%	2015 *n* = 33	%	2016 *n* = 25	%	2017 *n* = 19	%	2018 *n* = 22	%	2019 *n* = 59	%	2020 *n* = 77	%	2021 *n* = 40	%	2022 *n* = 42		2023 *n* = 54	%
*S. Enteritidis*	100	*S. Enteritidis*	45.45	*S. Enteritidis*	40	*S. Kentucky*	31.58	*S. Enteritidis*	50	*S. Enteritidis*	54.24	*S. Enteritidis*	18.18	*S. Enteritidis*	27.5	*S. Kentucky*	23.81	*S. Infantis*	24.07
		*S. Galli-narum*	45.45	*S. Typhi-* *murium*	32	*S. Derby*	21.05	*S. Typhi-* *murium*	10	*S. Indiana*	8.47	*S. Kentucky*	12.99	*S. Indiana*	15	*S. Typhi-* *murium*	21.43	*S. Enteritidis*	16.67
		*S. Indiana*	3.03	*S. Indiana*	16	*S. Enteritidis*	21.05	others	40	*S. Aba*	6.78	*S. Indiana*	10.39	*S. Typhi-* *murium*	10	*S. Enteritidis*	11.9	*S. Indiana*	14.81
		*S. Neften-bach*	3.03	*S. Hillingdon*	8	*S. Typhi-* *murium*	15.79			*S. Remo*	3.39	*S. Typhi-* *murium*	7.79	*S. Stanley*	5	*S. Corvallis*	7.14	*S. Kentucky*	11.11
		*S. Null*	3.03	*S. II*	4	*S. Infantis*	5.26			*S. Tado*	1.69	*S. Wangata*	7.79	*S. London*	5	*S. Mban-daka*	7.14%	*S. Corvallis*	3.7
						*S. Muenster*	5.26			*S. Thompson*	1.69	*S. Newport*	7.79	*S. Kottbus*	5	*S. Indiana*	4.76	*S. Schwarzen-grund*	3.7
										*S. Typhi-* *murium*	1.69	*S. Seremban*	6.49	*S. Rissen*	5	*S. Angoda*	2.38	*S. Typhi-* *murium*	3.7
										*S. Maghera-felt*	1.69	*S. Corvallis*	2.6	*S. Saintpaul*	5	*S. Infantis*	2.38	*S. Fresno*	1.85
										*S. Man-chester*	1.69	*S. Thompson*	2.6	*S. Kingston*	5	others	26.13	*S. Newport*	1.85
										*S. Chincol*	1.69	*S. Haifa*	2.6	*S. Agona*	2.5			others	18.34
										*S. Goettingen*	1.69	*S. Chincol*	1.3	Others	15				
										Others	15.25	*S. Shubra*	1.3						
												*S. Stanleyville*	1.3						
												*S. London*	1.3						
												Others	15.59						

### 3.2 Drug sensitivity test results

The resistance of *Salmonella* strains originating from swines and broilers to 14 antimicrobials across 10 classes from 2014 to 2023 is presented in Tables 3, 4. In the past ten years, the AMR of *Salmonella* from these sources has remained notably high. During the period of 2014–2023, the percentage of *Salmonella* strains from swines that exhibited resistance to at least one antimicrobial drug was as follows: 100.00, 100.00, 100.00, 87.10, 92.31, 92.42, 94.87, 77.78, 88.37, and 91.30%. The percentage of *Salmonella* strains from broilers resistant to at least one antimicrobial agent during in the same period was reported as 100.00, 93.34, 96.00, 94.74, 95.45, 83.05, 85.71, 77.50, 95.24, and 98.15%. Overall, there has been a general downward trend in the proportion of strains from both sources exhibiting resistance (≥ 1 resistant).

**TABLE 3:** Drug resistance results of 381 strains of *Salmonella* from swines to 14 antimicrobials, 2014–2023.

Year	Antimicrobial resistance in swine (*r*/n^1^pooled| prevalence^2^| 95% CI^2^)														
	**AM**			**A/C**			**GM**			**SPT**			**TE**		
2014	5/5	100.00	54.92–100	2/5	40.00	52.75–85.34	4/5	80.00	28.36–99.49	1/5	20.00	0.50–71.64	3/5	60.00	14.63–94.72
2015	13/16	81.25	54.35–95.95	10/16	62.50	35.43–84.80	4/16	25.00	7.26–52.38	7/16	43.75	19.75–70.12	16/16	100.00	82.92–100
2016	21/25	84.00	63.92–95.46	23/25	92.00	73.97–99.01	8/25	32.00	14.94–53.50	9/25	36.00	17.97–57.48	24/25	96.00	79.65–99.89
2017	22/31	70.97	51.96–85.78	4/31	12.90	3.63–29.83	17/31	54.84	36.03–72.68	26/31	83.87	66.27–94.55	23/31	74.19	55.39–88.14
2018	23/26	88.46	69.84–97.55	18/26	69.23	48.21–85.67	13/26	50.00	29.93–70.07	24/26	92.31	74.87–99.05	24/26	92.31	74.87–99.05
2019	50/66	75.76	63.64–85.46	10/66	15.15	7.51–26.10	16/66	24.24	14.54–36.36	37/66	56.06	43.29–68.26	59/66	89.39	79.36–95.63
2020	62/78	79.49	68.84–87.80	0/78	0.00	0.00–3.77	13/78	16.67	9.18–26.81	65/78	83.33	73.19–90.82	61/78	78.21	67.41–86.76
2021	29/45	64.44	48.78–78.13	27/45	60.00	44.33–74.30	16/45	35.56	21.87–51.22	31/45	63.89	53.35–81.83	35/45	77.78	62.91–88.79
2022	31/43	72.09	56.33–84.67	16/43	37.21	22.98–53.27	7/43	16.28	25.13–80.78	29/43	67.44	51.46–80.92	34/43	79.07	63.96–89.96
2023	35/46	76.09	61.23–87.41	19/46	41.30	26.99–56.77	4/46	8.70	2.42–20.79	22/46	47.83	32.89–63.05	36/46	78.26	63.64–89.05
Overall	291/381	76.38	71.79–80.55	129/381	33.86	29.12–38.85	102/381	26.77	22.39–31.52	281/381	73.75	69.03–78.10	315/381	82.68	78.50–86.34
**Year**	**FFC**			**SF**			**SXT**			**XNL**			**ENR**		
2014	4/5	80.00	28.36–99.49	3/5	60.00	14.66–94.73	1/5	20.00	0.50–71.64	0/5	0.00	0.00–45.07	1/5	20.00	0.50–71.64
2015	11/16	68.75	41.34–88.98	13/16	81.25	54.35–95.95	16/16	100.00	82.92–100	9/16	56.25	29.88–80.25	10/16	62.50	35.43–84.80
2016	14/25	56.00	34.93–75.60	23/25	92.00	73.97–99.02	13/25	52.00	31.31–72.20	5/25	20.00	6.83–40.70	8/25	32.00	14.95–53.50
2017	18/31	58.06	34.93–75.60	22/31	70.97	51.96–85.78	18/31	58.06	39.08–75.45	0/31	0.00	0.00–9.21	4/31	12.90	3.63–29.83
2018	15/26	57.69	36.92–76.65	23/26	88.46	69.84–97.55	17/26	65.38	44.33–82.79	12/26	46.15	26.59–66.63	3/26	11.54	2.45–30.15
2019	38/66	57.58	44.79–69.66	52/66	78.79	66.98–87.89	34/66	51.52	38.88–64.01	5/66	7.58	2.51–16.80	15/66	22.73	13.31–34.70
2020	59/78	75.64	64.60–84.65	68/78	87.18	77.68–93.68	60/78	76.92	66.00–85.71	0/78	0.00	0.00–3.77	18/78	23.08	14.29–34.00
2021	24/45	53.33	37.87–68.34	36/45	80.00	65.40–90.42	29/45	64.44	48.78–78.13	4/45	8.89	2.48–21.22	3/45	6.67	1.40–18.27
2022	25/43	58.14	42.12–72.99	32/43	74.42	58.83–86.48	25/43	58.14	42.13–72.99	4/43	9.30	2.59–22.14	1/43	2.33	0.05–12.29
2023	26/46	56.52	41.11–71.07	39/46	84.78	71.13–93.66	18/46	39.13	25.09–54.63	5/46	10.87	3.62–23.57	8/46	17.39	7.82–31.42
Overall	225/381	59.06	53.93–64.04	311/381	81.63	77.37–85.39	231/381	60.63	55.53–65.57	44/381	11.55	8.52–15.19	71/381	18.64	14.85–22.92
**Year**	**OFX**			**CL**			**CAZ**			**AP**					
2014	1/5	20.00	0.50–71.64	1/5	20.00	0.50–71.64	/	/	/	0/5	0	0–45.07			
2015	10/16	62.50	35.43–84.80	4/16	25.00	7.27–52.38	/	/	/	0/16	0	0–17.08			
2016	8/25	32.00	14.95–53.50	1/25	4.00	0.10–20.35	0/25	0	0–11.29	0/25	0	0–11.29			
2017	5/31	16.13	5.45–33.72	0/31	0.00	0.00–9.21	0/31	0	0–9.21	0/31	0	0–9.21			
2018	1/26	3.85	0.97–19.64	0/26	0.00	0.00–10.88	0/26	0	0–10.88	0/26	0	0–10.88			
2019	6/66	9.09	3.41–18.74	0/66	0.00	0.00–4.44	0/66	0	0–4.44	0/66	0	0–4.44			
2020	12/78	15.38	8.21–25.33	1/78	1.28	0.03–6.94	0/78	0	0–3.77	0/78	0	0–3.77			
2021	1/45	2.22	0.06–11.77	2/45	4.44	0.54–15.15	0/45	0	0–6.44	0/45	0	0–6.44			
2022	1/43	2.33	0.05–12.29	0/43	0.00	0.00–6.73	4/43	9.3	2.59–22.14	0/43	0	0–6.73			
2023	3/46	6.52	1.37–17.90	0/46	0.00	0.00–6.30	4/46	8.7	2.42–20.80	0/46	0	0–6.30			
Overall	48/381	12.59	9.44–16.35	9/381	2.36	1.09–4.44	0/381	2.10	0.91–4.10	0/381	0	0–0.78			

^1^*r* = represents the number of samples that tested positive for each antibiotic test; *n* = denotes the total number of samples used to assess resistance to each antibiotic.

^2^Analytical estimation was conducted using Minitab.

**TABLE 4:** Drug resistance results of 416 broiler-derived *Salmonella* strains to 14 antimicrobials, 2014–2023.

Year	Antimicrobial resistance in broiler (*r*/n^1^pooled | prevalence^2^| 95% CI^2^)														
	**AM**			**A/C**			**GM**			**SPT**			**TE**		
2014	39/45	86.67	73.21–94.95	11/45	24.44	12.88–39.54	9/45	20.00	9.57–34.60	11/45	24.44	12.88–39.54	18/45	40.00	25.70–55.67
2015	10/33	30.30	15.59–48.71	1/33	3.03	0.08–15.76	2/33	6.06	0.74–20.23	16/33	48.48	30.80–66.46	16/33	48.48	30.80–66.46
2016	20/25	80.00	59.30–93.17	20/25	80.00	59.30–93.17	5/25	20.00	6.83–40.70	15/25	60.00	38.67–78.87	13/25	52.00	31.31–72.20
2017	17/19	89.47	66.86–98.70	4/19	21.05	6.05–45.57	4/19	21.05	6.05–15.57	13/19	68.42	43.45–87.42	11/19	57.89	33.50–79.75
2018	21/22	95.45	77.16–99.89	11/22	50.00	28.22–71.78	10/22	45.45	24.39–67.79	10/22	45.45	24.39–67.79	17/22	77.27	54.63–92.18
2019	46/59	77.97	65.24–87.71	6/59	10.17	3.82–20.83	22/59	37.29	25.04–50.85	28/59	47.46	34.30–60.88	26/59	44.07	31.16–57.60
2020	48/77	62.34	50.56–73.13	5/77	6.49	2.14–14.51	26/77	33.77	23.38–45.45	33/77	42.86	31.63–54.65	52/77	67.53	55.90–77.77
2021	22/40	55.00	38.49–70.74	20/40	50.00	33.80–66.20	13/40	32.50	18.57–49.13	11/40	27.50	14.01–43.89	21/40	52.50	36.13–68.49
2022	33/42	78.57	63.19–89.70	12/42	28.57	15.72–44.58	18/42	42.86	27.72–59.04	27/42	64.29	48.03–78.45	24/42	57.14	40.96–72.28
2023	47/54	87.04	75.10–94.63	18/54	33.33	21.09–47.47	23/54	42.59	29.23–56.79	37/54	68.52	54.45–80.48	44/54	81.48	68.57–90.75
Overall	303/416	72.84	68.29–77.06	108/416	25.96	21.81–30.46	132/416	31.73	27.28–36.44	201/416	48.32	43.42–53.24	242/416	58.17	53.27–62.96
**Year**	**FFC**			**SF**			**SXT**			**XNL**			**ENR**		
2014	4/45	8.89	2.47–21.22	31/45	68.89	53.35–81.83	11/45	24.44	12.88–39.54	11/45	24.44	12.88–39.54	5/45	11.11	3.71–24.05
2015	4/33	12.12	3.40–28.20	24/33	72.73	54.48–86.70	29/33	87.88	71.80–96.60	3/33	9.09	1.92–24.33	2/33	6.06	0.74–20.23
2016	5/25	20.00	6.83–40.70	19/25	76.00	54.87–90.64	10/25	40.00	21.13–61.34	5/25	20.00	6.83–40.70	5/25	20.00	6.83–40.70
2017	7/19	36.84	16.29–61.64	15/19	78.95	54.43–93.95	10/19	52.63	28.86–75.55	4/19	21.05	6.05–45.57	4/19	21.05	6.05–45.57
2018	8/22	36.36	17.20–59.34	20/22	90.91	70.84–98.88	6/22	27.27	10.73–50.22	13/22	59.09	36.35–79.29	9/22	40.91	20.71–63.65
2019	25/59	42.37	29.61–55.93	41/59	69.49	56.14–80.81	24/59	40.68	28.07–54.25	19/59	32.20	20.62–45.64	23/59	38.98	26.55–52.56
2020	38/77	49.35	37.76–61.00	51/77	66.23	54.55–76.62	24/77	31.17	21.10–42.74	27/77	35.06	24.53–46.78	27/77	35.06	24.53–46.78
2021	16/40	40.00	24.87–56.67	15/40	37.50	22.73–54.20	9/40	22.50	10.84–38.45	11/40	27.50	14.60–43.89	9/40	22.50	10.84–38.45
2022	24/42	57.14	40.96–72.28	34/42	80.95	65.88–91.40	19/42	45.24	29.85–61.33	24/42	57.14	40.96–72.27	17/42	40.48	25.62–56.71
2023	40/54	74.07	60.35–85.04	42/54	77.78	64.40–87.96	30/54	55.56	41.40–69.08	30/54	55.56	41.4–69.08	13/54	24.07	13.49–37.64
Overall	171/416	41.10	36.34–46.01	292/416	70.19	65.54–74.55	172/416	41.35	36.57–46.25	147/416	35.34	30.74–40.14	114/416	27.40	23.17–31.96
**Year**	**OFX**			**CL**			**CAZ**			**AP**					
2014	3/45	6.67	1.40–18.27	37/45	82.22	67.95–92.00	/	/	/	0/45	0	0–6.44			
2015	2/33	6.06	0.74–20.23	18/33	54.55	36.35–71.89	/	/	/	0/33	0	0–8.68			
2016	4/25	16.00	4.54–36.08	9/25	36.00	17.97–57.48	3/25	12.00	2.55–31.22	0/25	0	0–11.29			
2017	3/19	15.79	3.38–39.58	0/19	0.00	0.00–14.59	4/19	21.05	6.05–45.57	0/19	0	0–14.58			
2018	9/22	40.91	20.71–63.65	10/22	45.45	24.39–67.79	5/22	22.73	7.82–45.37	0/22	0	0–12.73			
2019	24/59	40.68	28.07–54.25	0/59	0.00	0.00–4.95	19/59	32.20	20.62–45.63	0/59	0	0–4.44			
2020	21/77	27.27	17.74–38.62	7/77	9.09	3.73–17.83	17/77	22.08	13.42–32.98	0/77	0	0–3.81			
2021	9/40	22.50	10.84–38.45	6/40	15.00	5.71–29.84	11/40	27.50	14.60–43.89	0/40	0	0–7.21			
2022	14/42	33.33	19.57–49.55	1/42	2.38	0.06–12.57	13/42	30.95	17.62–47.09	0/42	0	0–6.88			
2023	11/54	20.37	10.63–33.53	2/54	3.70	0.45–12.75	11/54	20.37	10.63–33.53	0/54	0	0–5.40			
Overall	100/416	24.04	20.01–28.44	90/416	21.63	17.78–25.91	83/416	19.95	16.22–26.12	0/416	0	0–0.72			

^1^*r* = represents the number of samples that tested positive for each antibiotic test; *n* = denotes the total number of samples used to assess resistance to each antibiotic.

^2^Analytical estimation was conducted using Minitab.

In the past decade, *Salmonella* isolates from swines and broilers showed significant resistance to ampicillin, tetracycline, and sulfisoxazole, with resistance rates of 76.38% (291/381) and 72.84% (303/416) for ampicillin, 82.68% (315/381) and 58.17% (242/416) for tetracycline, and 81.63% (311/381) and 70.19% (292/416) for sulfisoxazole, respectively. Notably, *Salmonella* isolates from swines demonstrated a higher resistance rate to spectinomycin at 73.75% (281/381), whereas those from broilers exhibited a lower resistance rate of 48.32% (201/416), with a statistically significant difference (*P* < 0.01). The resistance profiles of *Salmonella* isolates to the various antimicrobials tested varied. Specifically, the drug resistance rates to two cephalosporins (ceftiofur and ceftazidime) were in descending order, while the drug resistance rate of isolates from swines and broilers to meropenem was 0%. The drug resistance rates to two aminoglycosides (spectinomycin and gentamicin) were also ranked in descending order. Furthermore, the drug resistance rates of the two sulfonamides were higher than those observed for methotrexate and gentamicin, as well as higher than those for metribuzin, metronidazole, and gentamicin. Finally, the drug resistance rates to the two quinolones (enrofloxacin and ofloxacin) were largely similar.

The drug resistance of *Salmonella* from various animal sources to different antimicrobial drugs exhibited significant variation across the years. In 2015, the resistance rate of *Salmonella* from swines (56.25%) to ceftiofur was notably higher than that of *Salmonella* from broilers (9.09%) (*P* < 0.01). However, in the subsequent years, the drug resistance rates of *Salmonella* strains from broilers to third-generation cephalosporins (ceftiofur, ceftazidime) were significantly higher (37.60%, 24.56%) compared to those from swines (13.20%, 2.22%) (*P* < 0.01). The drug resistance rate of *Salmonella* from swines to ceftazidime increased from 0 during 2016–2021 to 9.30% in 2022 and 8.70% in 2023, while the resistance rate of *Salmonella* from broilers to ceftazidime remained at around 25%. Furthermore, the resistance rate of *Salmonella* from broilers to mucin (21.63%) was significantly higher than that from swines (2.36%) (*P* < 0.01). The resistance rate of *Salmonella* from broilers to mucin varied considerably over the years, with 37 out of 45 isolates exhibiting mucin resistance in 2014, resulting in a high rate of 82.22%, followed by 54.55% in 2015. Over the next eight years, the mucin resistance rate in *Salmonella* from broilers decreased to 10.36%. In contrast, the overall resistance rate of *Salmonella* from swines to mucin remained very low, which was 2.26% (9/381). In 2015, only 4 of 16 isolates were resistant to mucin, and the tendency toward zero resistance in subsequent years. Additionally, in 2014, 2015, and 2016, *Salmonella* from swines demonstrated a higher resistance rate to enrofloxacin and ofloxacin than *Salmonella* from broilers. In the remaining seven years, however, the reverse was opposite.

Over the past decade, the drug resistance rate of swine-derived *Salmonella* to 14 antibacterial drugs was on the rise: spectinomycin and ceftazidime. The drug resistance rates have stabilized for tetracycline, fosfomycin, sulfamethoxazole, and meropenem. Conversely, the drug resistance rates decreased for ampicillin, gentamicin, enrofloxacin, ofloxacin, and mucomycin. Additionally, the drug resistance rates fluctuated for three drugs: amoxicillin-clavulanic acid, methotrexate/sulfamethoxazole, and ceftiofur. Amoxicillin-clavulanic acid, methotrexate/sulfamethoxazole, and ceftiofur were the three drugs with the most variable changge in drug resistance rates. The drugs exhibiting increasing resistance rates to 14 antimicrobials in *Salmonella* from broilers included ampicillin, gentamicin, macrolides, tetracycline, fosfomycin, ceftiofur, and enrofloxacin. In contrast, the drugs with stabilizing resistance rates were ceftazidime and meropenem. In addition, ofloxacin and mucomycin demonstrated decreasing resistance rate. The three drugs with the most variable resistance rates were amoxicillin-clavulanic acid, sulfisoxazole, and methotrexate/sulfamethoxazole.

The multi-resistance of *Salmonella* originating from swines and broilers to the 10 classes of antimicrobials tested from 2014 to 2023 is illustrated in Tables 5, 6. Both *Salmonella* strains exhibited significant multi-resistance, with multi-drug resistance rate (MDR) of 80.58% (307/381) and 70.67% (294/416), respectively (*P* < 0.01). The MDR of *Salmonella* from swines reached a peak (92.30%) in 2016 and the lowest point (68.89%) in 2021. In contrast, *Salmonella* from broilers demonstrated the highest MDR in 2018, accounting for 95.45%, followed by lower MDR in 2019, which was 50.85 and 50.00% in 2021.

**TABLE 5:** Multi-drug resistance results for *Salmonella* from swines, 2014–2023.

Year	2014	2015	2016	2017	2018	2019	2020	2021	2022	2023	Overall
Number of isolates tested	5	16	25	31	26	66	78	45	43	46	381
Resistance pattern											
No resistance detected	0	0	0	4 (12.9%)	2 (7.69%)	5 (7.57%)	4 (5.13%)	10 (22.22%)	5 (11.63%)	4 (8.7%)	34 (8.92%)
Resistance = 1 CLSI Class^1^	1 (20%)	0	1 (4%)	4 (12.9%)	0	5 (7.58%)	2 (2.56%)	3 (6.67%)	2 (4.65%)	3 (6.52%)	21 (5.51%)
Resistance = 2 CLSI Classes^1^	0	2 (12.5%)	1 (4%)	1 (3.23%)	0	4 (6.06%)	4 (5.13%)	1 (2.22%)	5 (11.63%)	1 (2.17%)	19 (4.99%)
Resistance = 3 CLSI Classes^1^	0	0	0	0	1 (3.85%)	9 (13.64%)	7 (8.97%)	3 (6.67%)	3 (6.98%)	4 (8.7%)	27 (7.09%)
Resistance = 4 CLSI Classes^1^	0	3 (18.75%)	9 (36%)	4 (12.9%)	3 (11.54%)	8 (12.12%)	13 (16.67%)	4 (8.89%)	3 (6.98%)	9 (19.57%)	56 (14.7%)
Resistance = 5 CLSI Classes^1^	2 (40%)	1 (6.25%)	1 (4%)	11 (35.48%)	5 (19.23%)	17 (25.76%)	34 (43.59%)	6 (13.33%)	11 (25.58%)	13 (28.26%)	101 (26.51%)
Resistance = 6 CLSI Classes^1^	1 (20%)	0	6 (24%)	6 (19.35%)	3 (11.54%)	15 (22.73%)	14 (17.95%)	9 (20%)	12 (27.91%)	10 (21.74%)	76 (19.95%)
Resistance = 7 CLSI Classes^1^	0	7 (43.75%)	7 (28%)	1 (3.23%)	11 (42.31%)	3 (4.55%)	0	7 (15.56%)	2 (4.65%)	2 (4.35%)	40 (10.5%)
Resistance = 8 CLSI Classes^1^	1 (20%)	1 (6.25%)	0	0	1 (3.85%)	0	0	2 (4.44%)	0	0	5 (1.31%)
Resistance = 9 CLSI Classes^1^	0	2 (12.5%)	0	0	0	0	0	0	0	0	2 (0.52%)

^1^CLSI, Clinical and Laboratory Standards Institute.

**TABLE 6:** Multi-drug resistance results for *Salmonella* from broilers, 2014–2023.

Year	2014	2015	2016	2017	2018	2019	2020	2021	2022	2023	Overall
Number of isolates tested	45	33	25	19	22	59	77	40	42	54	416
Resistance pattern											
No resistance detected	0	2 (6.06%)	1 (4%)	1 (5.26%)	1 (4.55%)	10 (16.95%)	11 (14.28%)	9 (22.5%)	2 (4.76%)	1 (1.85%)	38 (9.13%)
Resistance = 1 CLSI Class 1	3 (6.67%)	1 (3.03%)	2 (8%)	0	0	2 (3.39%)	8 (10.39%)	5 (12.5%)	5 (11.9%)	1 (1.85%)	27 (6.49%)
Resistance = 2 CLSI Classes^1^	5 (11.11%)	2 (6.06%)	1 (4%)	4 (21.05%)	0	17 (28.81%)	8 (10.39%)	6 (15%)	5 (11.9%)	9 (16.67%)	57 (13.7%)
Resistance = 3 CLSI Classes^1^	5 (11.11%)	16 (48.48%)	1 (4%)	4 (21.05%)	2 (9.09%)	5 (8.47%)	13 (16.88%)	4 (10%)	3 (7.14%)	3 (5.56%)	56 (13.46%)
Resistance = 4 CLSI Classes^1^	19 (42.22%)	9 (27.27%)	5 (20%)	4 (21.05%)	5 (22.73%)	2 (3.39%)	3 (3.9%)	2 (5%)	2 (4.76%)	3 (5.56%)	54 (12.98%)
Resistance = 5 CLSI Classes^1^	4 (8.89%)	1 (3.03%)	9 (36%)	2 (10.53%)	4 (18.18%)	1 (1.69%)	7 (9.09%)	3 (7.5%)	1 (2.38%)	7 (12.96%)	39 (9.38%)
Resistance = 6 CLSI Classes^1^	6 (13.33%)	0	2 (8%)	0	3 (13.64%)	3 (5.08%)	11 (14.29%)	0	13 (30.95%)	14 (25.93%)	52 (12.5%)
Resistance = 7 CLSI Classes^1^	1 (2.22%)	2 (6.06%)	4 (16%)	0	3 (13.64%)	16 (27.12%)	12 (15.58%)	2 (5%)	7 (16.67%)	10 (18.52%)	57 (13.7%)
Resistance = 8 CLSI Classes^1^	2 (4.44%)	0	0	4 (21.05%)	2 (9.09%)	3 (5.08%)	4 (5.19%)	1 (2.5%)	4 (9.52%)	6 (11.11%)	26 (6.25%)
Resistance = 9 CLSI Classes^1^	0	0	0	0	2 (9.09%)	0	0	8 (20%)	0	0	10 (2.4%)

^1^CLSI, Clinical and Laboratory Standards Institute.

**FIGURE 2:**
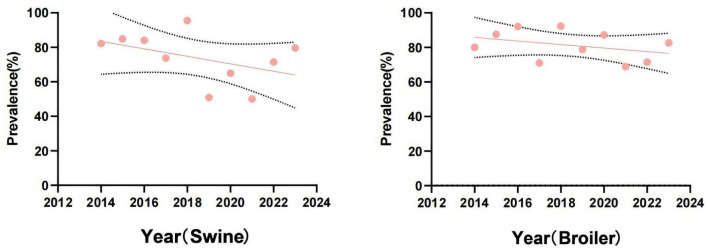
Trends in multi-drug resistance of *Salmonella* from swines and broilers, 2014–2023.

The MDR of *Salmonella* isolates from swines and broilers showed an overall decreasing trend during the last 10 years (Figure 2). The problem of high MDR of broiler-origin *Salmonella* compared with swine-origin *Salmonella* was prominent. 5drug-resistant and above strains were 123 (32.28%) and 123 (34.85%), respectively, *p* > 0.05; 8drug-resistant and above strains were found in total 7 (1.83%) and 36 (8.65%), respectively, *p* < 0.01, and no 10 drug-resistant strains were found.

### 3.3 Detection results of ESBLs-producing strains

**FIGURE 3:**
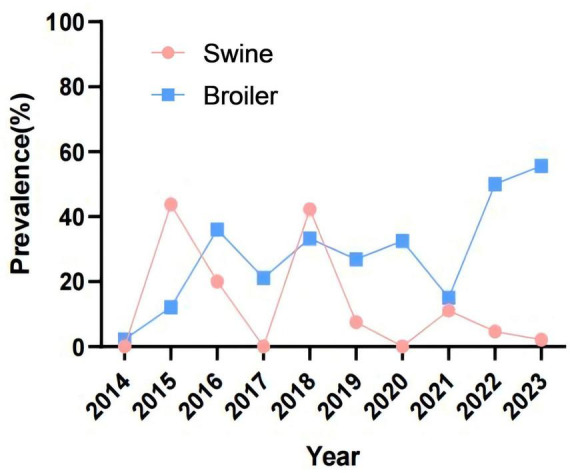
Detection of *Salmonella*-produced ESBLs from swines and broilers, 2014–2023.

The results about ESBLs-producing *Salmonella* strains were shown in Figure 3. Out of the 381 strains of *Salmonella* from swines, 36 were identified as ESBL-producers, resulting in a detection rate of 9.45%. It is worth noting that no ESBL-producing strains were detected in 2014, 2017, and 2020, indicating an overall decreasing trend in the detection rate of ESBL-producing *Salmonella* from swines in the past decade. In contrast, 126 ESBL-producing strains were identified from broilers, yielding a detection rate of 29.58%. The detection rate surpassed 50% in both 2022 and 2023, reflecting a significant increasing trend in the prevalence of ESBL-producing *Salmonella* from broilers over the last 10 years.

### 3.4 Multilocus sequence typing (MLST)

**FIGURE 4:**
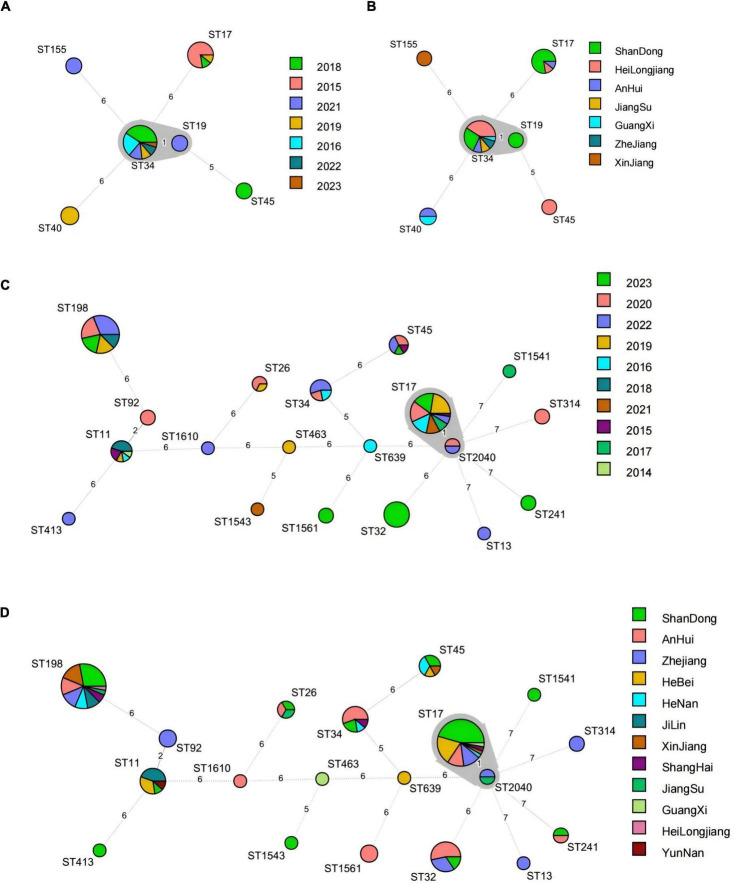
MLST minimum spanning tree based on year and province for *Salmonella* ESBLs from swines and broilers. **(A)** MLST minimum spanning tree based on year for 36 swine-derived ESBLs *Salmonella* strains; **(B)** MLST minimum spanning tree based on province for 36 swine-derived ESBLs *Salmonella* strains; **(C)** MLST minimum spanning tree based on year for 126 broiler-derived ESBLs *Salmonella* strains. **(D)** MLST minimum spanning tree based on province for 126 broiler-derived ESBLs *Salmonella* strains.

The MLST typing results referred to ESBL-producing *Salmonella* isolates, along with the MLST minimum spanning tree constructed based on the year and province of swine and broiler-origin *Salmonella*, were presented in Figure 4. A total of 6 ST types were identified among the 36 swine-origin ESBL-producing *Salmonella* isolates, with the dominant types being ST34 (61.11%) and ST17 (25.00%). Specifically, ST34 isolates were detected over 6 years (2016, 2018, 2019, 2021–2023) and across 6 provinces (Heilongjiang, Shandong, Anhui, Jiangsu, Zhejiang, and Guangxi). These isolates differed from ST19 by 1 allele, all of which were classified as *S. Typhimurium*. In contrast, ST17 isolates were identified in 3 provinces (Shandong, Heilongjiang, and Anhui) within 3 years (2015, 2018, and 2019) In total, ST19 types were identified in broiler-derived ESBL *Salmonella*. The dominant ST types included ST17 (27.78%), ST198 (24.60%), and ST32 (9.52%). Among these, ST17 isolates were present over 8 years (2015–2017, 2019–2023) and across 8 provinces (Shandong, Hebei, Heilongjiang, Zhejiang, Henan, Anhui, Shanghai, and Guangxi), differing from ST2040 by 1 allele, both of which were classified as *S. Indiana*. ST198 isolates were detected over 5 years (2018–2020, 2022, and 2023) and across 9 provinces (excluding Hebei, Guangxi, and Yunnan). Finally, ST32 isolates were all obtained in 2023, and distributed in 3 provinces (Anhui, Zhejiang, and Shandong). Notably, ST17, ST34, and ST45 were ST types shared between *Salmonella* of swine and broiler origin.

### 3.5 Core genome multilocus sequence typing (cgMLST)

**FIGURE 5:**
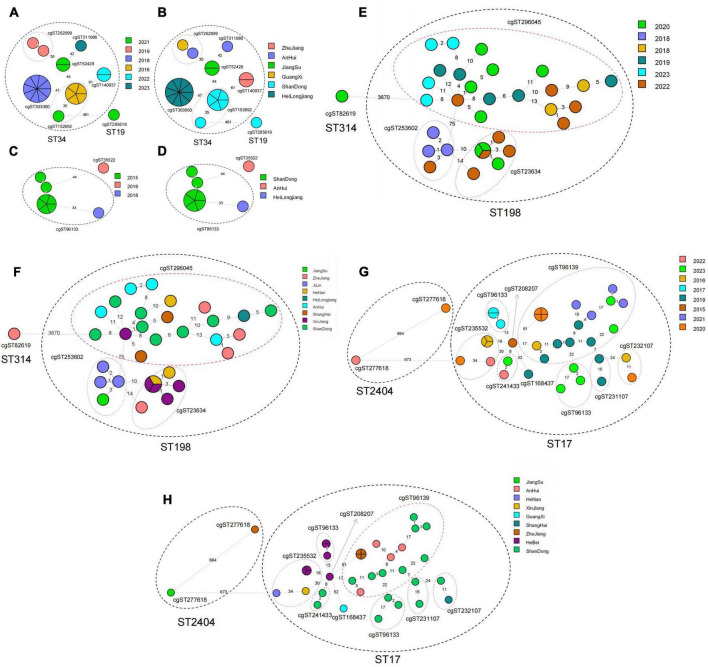
Minimum generation tree of cgMLST type based on year and province for different serotypes of *Salmonella* produced ESBLs from swines and broilers. **(A)** Year-based cgST-type minimum spanning tree for ESBLs *S. Typhimurium* (ST34, ST19) from swines. **(B)** Province-based cgST-type minimum spanning tree for ESBLs *S. Typhimurium* (ST34, ST19) from swines. **(C)** Year-based cgST-type minimum spanning tree for ESBLs *S. Indiana* (ST17) from swines. **(D)** Year-based cgST-type minimum spanning tree for Province-based cgST-type minimum spanning tree for ESBLs *S. Indiana* (ST17). **(E)** Year-based cgST-type minimum spanning tree for ESBLs *S. Indiana* (ST17, ST2404) from broilers. **(F)** Province-based cgST-type minimum spanning tree for ESBLs *S. Indiana* (ST17, ST2404) from broilers. **(G)** broiler-derived ESBLs *S. Kentucky* (ST198, ST314) year-based cgST-type minimum spanning tree; **(H)** broiler-derived ESBLs *S. Kentucky* (ST198, ST314) province-based cgST-type minimum spanning tree.

The cgMLST minimum generation trees of the dominant serotypes of *S. Typhimurium* and *S. Indiana*, based on year and province for swine-produced ESBLs from 2014 to 2023, were illustrated in Figures 5A–D. A total of 23 strains of *S. Typhimurium* were classified into ST34 (22 strains) and ST19 (1 strain). The ST34 strains were further divided into 6 cgST types, with differences ranging from 0 to 61 loci between strains. The dominant cgST types were cg303060 (9 strains) and cg152692 (6 strains). It is worth noting that all cg303060 strains came from Heilongjiang Province in 2018, while cg152692 strains came from Shandong Province in 2016 and 2021 Additionally, 9 strains of *S. Indiana* (ST17) were categorized into 2 cgST types, with different ranging from 0 to 44 loci between strains. The predominant cgST type was cg96133 (8 strains), which included 7 strains from Shandong Province in 2015 and 1 strain from Heilongjiang Province in 2018.

The minimum generation trees for the dominant serotypes of *S. Indiana* and *S. Kentucky*, based on year and province for broiler-derived ESBLs from 2014 to 2023, were illustrated in Figures 5E–H. A total of 37 strains of *S. Indiana* were categorized into ST17 (35 strains) and ST2040 (2 strains). Within ST17, there were 9 cgST types identified, with strain differences ranging from 0 to 61 loci. The predominant cgST type was cg96139, which included 8 strains from Shandong Province collected in 2016, 2019, 2021, and 2023, 4 strains from Anhui Province in 2019 and 2021, and 4 strains from Zhejiang Province in 2020. Notably, cg96139 exhibited higher homology with cg23107, cg96133, cg208207 and cg41433. Meanwhile, the 33 strains of *S. Kentucky* were classified into ST198 (32 strains) and ST314 (1 strain). ST198 was further divided into three cgST types, with differences between strains ranging from 0 to 71 loci. The dominant cgST type was 296405, which included 20 strains, comprising 8 strains from Shandong Province in 2019, 2020, 2022, and 2023, 4 strains from Anhui Province in 2018 and 2023, 1 strain from Heilongjiang Province in 2018, 1 strain from the Xinjiang Uygur Autonomous Region in 2022, and 4 strains from Henan Province (1 strain), Shanghai Province (2 strains), and Zhejiang Province (1 strain) in 2020, as well as 2 strains from Zhejiang Province in 2022. Among these, cg23634 and cg253692 demonstrated greater homology.

### 3.6 Drug resistance gene comparison results

**FIGURE 6:**
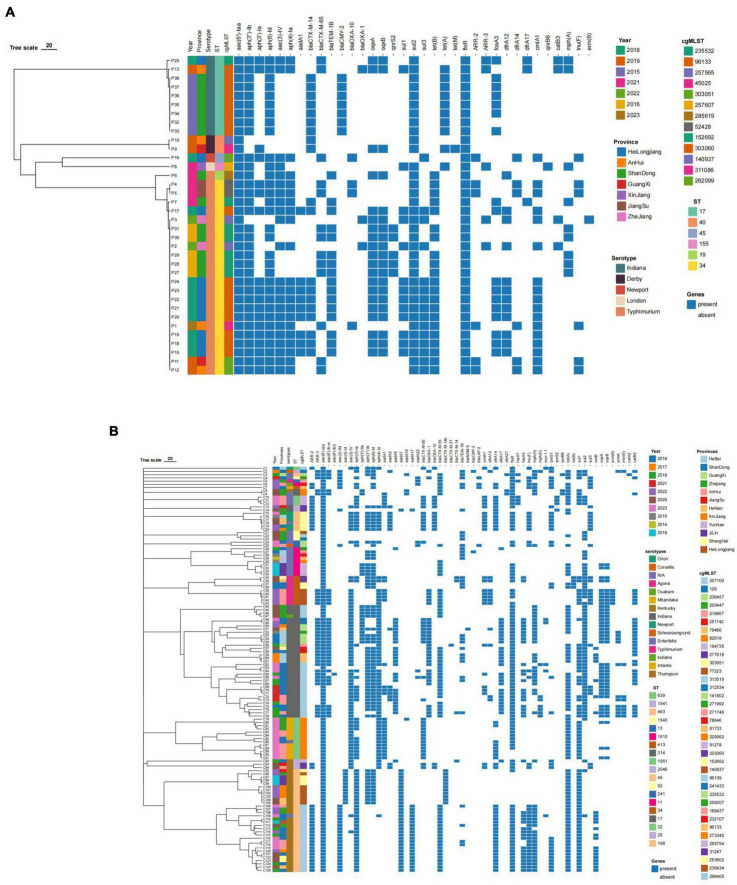
Heatmap of resistance genes of *Salmonella* strains from swines and broilers. **(A)** Heat map of drug resistance genes of 36 swine-derived ESBLs-producing *Salmonella* strains. **(B)** Heat map of drug resistance genes of 126 broiler-derived ESBLs-producing *Salmonella* strains.

The results of resistance gene testing of 36 swine-derived ESBLs-producing *Salmonella* strains and 126 broiler-derived ESBLs-producing *Salmonella* strains from 2014 to 2023 were shown in Table 7 and Figure 6. The drug resistance phenotype of *Salmonella* produced in ESBLs from swines and broilers is consistent with the results of the resistance genes carried. A total of 35 drug resistance genes across 11 classes were identified in 36 swine-derived ESBL-producing *Salmonella* isolates. All isolates harbored antibiotic resistance genes corresponding to aminoglycosides (7), β-lactams (6), sulphonamides (3), and amidinols (3). Among these, the aminoglycoside resistance gene *aac(6*′*)-Iaa* and the amidinols resistance gene *floR* were detected in 100.00% of the isolates. The predominant β-lactam resistance gene subtype was *blaCTX-M-14* (20/36 isolates, 55.56%), followed by *blaTEM-1B* (16/36 isolates, 44.44%) and *blaCTX-M-65* (13/36 isolates, 36.11%). Additionally, the major resistance genes for the remaining eight classes of antimicrobials included *0qxA*/*0qxB*, *sul2*, *tet*(B), *ARR-2*, *fosA3*, *dfrA12*, *mph*(A), and *lnu*(F), with detection rates ranging from 16.67% [*lnu*(F)] to 86.11% [*tet*(B)]. Notably, no isolates were found to carry peptide mucin resistance genes. Among 126 strains of ESBL-producing *Salmonella* isolated from broilers, 52 kinds of drug resistance genes were identified in 12 classes, all of which harbored aminoglycoside (20) and β-lactam (11) drug resistance genes. Notably, the aminoglycoside resistance gene *aac(6*′*)-Iaa* was present in 100% of the isolates. The predominant β-lactam resistance gene isoform was *blaCTX-M-55* (64/126, 50.79%), followed by *blaCTX-M-65* (37/126, 29.36%), *blaTEM-1B* (32/126, 25.39%) and *blaOXA-1* (31/126, 24.60%). Additionally, the major resistance genes for the remaining 10 classes of antimicrobials included *0qxA/0qxB*, *sul1*, *tet*(A), *floR*, *ARR-2*, *fosA3*, *dfrA14*, *mph*(A), *lnu*(F) and *mcr-1.1*, with detection rates ranging from 4.76% (*mcr-1.1*) to 96.03% (*sul1*).

**TABLE 7:** Results of resistance gene testing of ESBLs-producing *Salmonella* isolates from swines and broilers, 2014–2023.

Class drug	Swine-derived drug resistance gene (Carrier Strain)	Detection rate	broiler-Derived Drug Resistance gene (Carrier Strain)	Detection rate
Aminoglycoside	*aac(6*′*)-Iaa* (36), *aph(3*″*)-Ib*(33), *aph(6)-Id*(32), *aph(3*′*)-Ia*(18), *aac(3)-IV*(22), *aph(4)-Ia*(21), *aadA1*(9)	100%	*aac(6*′*)-Iaa*(126), *aph(3*′*)-Ia*(70), *aac(3)-IV*(68), *aph(4)-Ia*(67), *aph(6)-Id*(66), *aph(3*″*)-Ib*(62), *aac(6*′*)-Ib-cr*(33), *aadA7*(33), *aac(3)-IId*(29), *aadA1*(28), *aadA5*(27), *aadA17*(22), *rmtB*(22), *aph(3*′*)-IIa*(18), *aadA2*(16), *armA*(12), *aac(3)-Id*(11), *aadA22*(7), *aadA16*(4), *aac(6*′*)-Ib3*(1)	100%
βeta-lactam	*blaCTX-M-14*(20), *blaTEM-1B*(16), *blaCTX-M-65*(13), *blaCMY-2*(7), *blaOXA-10*(4), *blaOXA-1*(3)	100%	*blaCTX-M-55*(64), *blaCTX-M-65*(37), *blaTEM-1B*(32), *blaOXA-1*(31), *blaCTX-M-14b*(11), blaOXA-10(10), *blaCTX-M-14*(4), *blaLAP-2*(4), *blaCMY-2*(2), *blaCTX-M-27*(1), *blaNDM-5*(1)	100%
Fluoroquinolone	*oqxA*(20), *oqxB*(20), *qnrS2*(5), *qnrB6*(1)	58.33%	*oqxA*(39), *oqxB*(39), *qnrS1*(33), *qnrS2*(4), *qnrB6*(3)	61.11%
Sulphonamide	*sul2*(35), *sul3*(15), *sul1*(13)	100%	*sul1*(78), *sul2*(62), *sul3*(25)	96.03%
Tetracycline	*tet*(B)(21), *tet*(A)(10), *tet*(M)(1)	86.11%	*tet(A)*(93), *tet(B)*(4)	75.40%
Amphenicols	*floR*(36), *cmlA1*(17), *catB3*(4)	100%	*floR*(101), catB3(31), *cmlA1*(25), *catA2*(1)	80.16%
Rifampicin	*ARR-2*(6), *ARR-3*(5)	30.56%	*ARR-2*(37), *ARR-3*(34)	57.14%
Fosfomycin	*fosA3*(17)	47.22%	*fosA3*(39), *fosA7*(5)	35.71%
Trimethoprim	*dfrA12*(12), *dfrA14*(6), *dfrA17*(3)	58.33%	*dfrA12*(12), *dfrA14*(45), *dfrA17*(28), *dfrA27*(3)	67.46%
Macrolide	*mph*(A)(9)	25%	*mph*(A)(53), *mph*(E)(8), *erm*(B)(5), *msr*(E)(8)	50.79%
Lincosamide	*lnu*(F)(6)	16.67%	*lnu*(F)(38)	69.84%
Peptides			*mcr-1.1*(6)	4.76%

## 4 Discussion

### 4.1 Popular trend of *Salmonella* originating from swines/broilers

*Salmonella* infections are a significant global public health issue, outbreaks of human *Salmonella* disease are often associated with certain serotypes of *Salmonella* originating from swines and broilers (Ferrari et al., 2019). According to research by Hendriksen et al. (2011) *S. Typhimurium* was the most common serotype in North America, Asia, Africa, and Oceania, followed by *S. Enteritidis*. The present study indicated that *S. Typhimurium* had been the most prevalent serotype among host swines in China over the past decade, maintaining dominance in all years, except for 2014, 2015 and 2019. In contrast, *S. Enterica* ranked sixth, further suggesting that swines were a primary vector of *S. Typhimurium* in China. Broilers can be infected with various *Salmonella* serotypes and, similar to swines, are typically asymptomatic carriers, with *S. Enterica* being the most prevalent serotype in host broilers and the most common serotype found in humans across Asia, Latin America, Africa, and Europe (Hendriksen et al., 2011). This study also demonstrated that broilers were the main carriers of *S. Enterica* in China, with *S. Enterica* being the dominant serotype in all years except for 2017, 2022 and 2023. *S. Pullorum* is a serotype that is unique to broilers and can lead to severe systemic diseases in these birds. This condition results in considerable economic losses for the broiler industry, stemming from direct losses, costs associated with flock replacement, and treatment expenses (Farhat et al., 2023). This study indicated that no isolates of *Salmonella* fowl dysentery have been detected in China over the past decade, and only in 2015 were 15 isolates of fowl typhoid identified, all from the same farm, without a diffuse distribution. This decline could be attributed to China’s recent attention to the purification of animal diseases, as outlined in the Opinions on Promoting the Purification of Animal Diseases, including avian salmonellosis, and the implementation of a protocol for the purification of *Salmonella* fowl dysentery in breeder farms. These measures had effectively prevented and controlled the spread and prevalence of *Salmonella* fowl dysentery.

Multi-Locus Sequence Typing (MLST), one of the common molecular typing methods, has been extensively utilized in *Salmonella* genotyping, often demonstrating a strong correlation between serotypes and sequence types (STs) (Elnekave et al., 2020). In this study, each serotype was predominantly associated with a single ST type. However, instances were noted where a single serotype was represented by two distinct ST types. For example, *S. Kentucky* was categorized into ST314 and ST198, while *S. Indiana* was divided into ST2040 and ST17. This suggested that MLST possessed a higher resolution than serotyping. Our findings indicated that the dominant strains of ESBL-producing *Salmonella* from swines were ST34 (*S. Typhimurium*), whereas the most common ESBL-producing strains from broiler origin were ST17 (*S. Indiana*) and ST198 (*S. Kentucky*). This implies that the three serotyped *Salmonella* isolates—*S. Typhimurium*, *S. Indiana*, and *S. Kentucky*—exhibited a greater susceptibility to producing ultra-broad-spectrum β-lactamase. In contrast, the prevalent *S. Enteritidis* strains from broiler origin were predominantly non-ESBL-producing. These results were consistent with the findings of Franceschine et al. (2023) and Yang et al. (2014). MLST cluster analysis revealed that certain *Salmonella* isolates from swines and broilers over the past decade were related; for instance, ST34 *Salmonella* isolates from swines spanned six years, while ST17 *Salmonella* isolates from broilers sources spanned eight years. This suggested that there was clonal transmission between *Salmonella* isolates of swines and broilers in different years. In recent years, with the continuous enhancement of traceability technology, core genome MLST (cgMLST) appeared as an innovative method for strain traceability analysis (Gu et al., 2020) and was widely applied in the traceability of *Salmonella* isolates. cgMLST is a high-resolution genome typing method based on the core genome, which can identify the differences of *Salmonella*’s core genome sequences. This study demonstrated that the ST34 swine-derived strain and the ST17 broiler-derived strain were classified into six and nine cgST types, respectively, revealing a genetic evolutionary relationship among strains of different cgST types. This indicated that strains with the same ST may either represented the same clone or exhibited genetic variation. Yan et al. (2021) found that cgMLST correlated well with the temporal and regional distribution of bacterial isolates. In this study, we also observed that a particular cgST type was detected over five consecutive years or only within one year. Notably, a cgST type that appeared annually over a ten-year period was not detected, which may be attributed to the mutation of bacterial genome over time. Furthermore, we found that one cgST type generally corresponded to a single province, with only a few instances of the same cgMLST strains identified across different provinces, potentially due to genomic mutations associated with regional changes. The results of microbial epidemiological investigations indicated that the predominant pathogens might shift during an outbreak, a phenomenon that was often overlooked by conventional low-resolution molecular typing methods (Quick et al., 2015; Yan et al., 2021). In this study, cgMLST successfully distinguished these variations, with no single cgST containing two or more serotypes. Additionally, cgMLST provided the ability to discern minor differences between isolates and clusters with significantly greater accuracy than MLST.

This study also had several shortcomings. While the selected provinces for sampling were representative, the sampling range didn’t comprehensively encompass the entirety of actual farming production. Consequently, the epidemiological data obtained were subject to certain limitations.

### 4.2 Resistance characteristics of *Salmonella* originating from swines/broilers

The results of the drug sensitivity tests in this study indicated that *Salmonella* isolates from swines and broilers had significant drug resistance to ampicillin, tetracycline, sulfisoxazole and macrolides. This drug resistance might be attributed to the long-term and extensive application of these four drugs in the clinical environment. Tetracycline and sulfonamides were introduced for veterinary use in the 1960s, while ampicillin and macrolides were adopted in the 1970s (Zhang et al., 2017). Furthermore, data from 2020 reveal that the three most commonly used veterinary antimicrobials in China were tetracyclines (30.52%), sulfonamides and synergists (13.08%) and β-lactams and inhibitors (12.55%). This trend indicated the correlation between prolonged and intensive antimicrobial usage and the emergence of high levels of drug resistance, ranging from 48.32 to 82.68%. The trend in the prevalence of *Salmonella* drug resistance over the past decade indicated a decrease in the resistance rates of swine- and broiler-derived strains to certain antimicrobial drugs. This decline might be attributed to a series of veterinary drug use management policies implemented by China in recent years concerning livestock and poultry farming. Specifically, Bulletin No. 2428 from the Ministry of Agriculture of the People’s Republic of China mandates the discontinuation of mucomycin for animal growth promotion as of 1 November 2016. Notably, mucomycin resistance in this study was significantly higher during the years 2014–2017 compared to 2017–2023. Whole Genome Sequencing (WGS) analyses revealed that *Salmonella* from swines did not harbor the mucomycin-resistant gene, *mcr-1.1*. In contrast, four strains from broilers identified in 2023 carried the gene on transposons and plasmids containing mobile genetic elements, which likely contributed to the observed increase in mucomycin resistance rates in 2023. Additionally, Bulletin No. 2292 from the Ministry of Agriculture of the People’s Republic of China prohibits the use of four fluoroquinolones, including ofloxacin, in food animals since 31 December 2016. In this study, we observed an overall decreasing trend in the resistance of *Salmonella* from swines to ofloxacin. Conversely, the resistance of *Salmonella* from broilers to ofloxacin exhibited an increasing trend from 2017 to 2018, stabilized from 2018 to 2019, and subsequently showed an overall decreasing trend from 2019 to 2023. To comprehensively address the issue of bacterial drug resistance, China initiated a pilot action to reduce the use of veterinary antimicrobials in 2018, with plans for nationwide implementation from 2021 to 2025. Zhao et al. (2024) analyzed data and found that the amount of antimicrobial drugs used per tons of animal products in China had been decreasing year by year since 2014. This decline might significantly contribute to the observed slowing and decreasing trend in resistance to certain antimicrobial drugs among *Salmonella* originating from swines and broilers, indicating that the series of veterinary drug management policy measures adopted in China are appropriate and effective. In addition, this study noted a similar decreasing trend in the detection rate of ESBLs-producing *Salmonella*, a key concern for the WHO, particularly among swine isolates, which exhibited a detection rate of 9.45%—significantly lower than the 29.58% observed in broiler-derived isolates. This difference might be attributed to enhanced biosecurity measures and epidemic monitoring implemented in regions following the outbreak of African swine fever in 2018, resulting in reduced use of veterinary antimicrobials on swine farms and subsequently curbing the emergence of drug-resistant bacteria. However, it is important to highlight the sudden increase in the detection rate of ESBLs-producing *Salmonella* from broilers in 2022 and 2023, indicating that the use of β-lactams in broiler farms remained prevalent during these years and warrants further attention. Fortunately, no *Salmonella* isolates resistant to carbapenem antibiotics were identified in the samples from swines and broilers in this study.

In this study, drug resistance genes of the strains producing extended-spectrum beta-lactamases (ESBLs) were analyzed (Schürch et al., 2018). It was observed that both swine-origin and broiler-origin strains harbored multiple resistance genes. Specifically, 35 resistance genes across 11 classes were detected in swine-origin strains, while 52 resistance genes across 12 classes were identified in broiler-origin strains. This coexistence of multiple resistance genes might have contributed to the significant problem of multi-drug resistance among *Salmonella* isolates from ESBLs-producing strains, aligning with previous findings that reported a high rate of multi-drug resistance in *Salmonella*. Furthermore, this supports the assertion that ESBLs-producing strains did pose a serious challenge to drug resistance, corroborating the results of the study conducted by Lee et al. (2019). Furthermore, this study indicated that ESBLs-producing *Salmonella* contained the highest number of aminoglycoside resistance genes, with counts of 7 and 20 for swine and broiler origins, respectively, demonstrating a broad distribution of resistance. This finding aligned with the results reported by Zhang et al. (2023), thereby elucidating the heightened resistance to aminoglycosides. Among the β-lactam resistance genes identified, the predominant types were *TEM* and *CTX-M*. *TEM-1*, one of the earliest reported *TEM*-type β-lactamases, was usually encoded by plasmids and exhibits resistance primarily to ampicillin. It was commonly found in Gram-negative bacteria, including *E. coli* and *Klebsiella pneumoniae* (Elumalai et al., 2014). Doosti et al. (2016) reported that 73.1% of *Salmonella* detected in poultry carcasses carried the *TEM-1* gene. In this study, *Salmonella* from swines and broilers exhibited the presence of *blaTEM-1B* at rates of 44.44 and 25.39%, respectively, indicating a widespread occurrence of *TEM-1* in *Salmonella*. *CTX-M*-type extended-spectrum β-lactamases (ESBLs) are generally categorized into 6 subgroups, with *CTX-M-1* and *CTX-M-9* being the most frequently reported. *CTX-M-15* and *CTX-M-14* represent the major gene isoforms within these subgroups, respectively. However, our study revealed that *Salmonella* of swine and broiler origin carried distinct predominant *CTX-M* genotypes. Specifically, *Salmonella* from swine sources primarily harbored *blaCTX-M-14* (55.56%) and *blaCTX-M-65* (36.11%), while strains from broiler sources predominantly carried *blaCTX-M-55* (50.79%) and *blaCTX-M-65* (29.36%). It was noteworthy that although no carbapenem-resistant strains were found in this study, the *blaNDM-5* gene was detected in *S. Indiana* isolate from broilers. The *blaNDM* gene encoded a carbapenemase that confers resistance to carbapenems and most other beta-lactam antimicrobial agents, representing a significant public health threat. The *blaNDM-5* variant was previously identified by Wang et al. (2020) in retail pork in China. Additionally, Wang et al. (2020) reported the presence of *S. Typhimurium* harboring *blaNDM-5* in retail pork, while Li et al. (2017) detected this gene in children’s feces, and Deng et al. (2024) found it in environmental samples, all within China. These findings suggested that *blaNDM-5* genes were widespread across various samples from animals, environmental sources, and humans in China, highlighting the need to consider the complexity and diversity of serotypes. Fosfomycin is an effective antimicrobial agent against both Gram-negative and Gram-positive bacteria. Although it is not approved for use in Chinese veterinary clinics, the present study revealed the presence of fosfomycin-resistant genes, *fosA7* (0 and 3.97%) and *fosA3* (47.22 and 30.95%), in swine and broiler-derived ESBL-producing *Salmonella* spp. In contrast, Wang et al. (2022) reported the detection of *fosA7* and *fosA3* in Chinese food animals at rates of 6.53 and 9.26%, respectively. This discrepancy might be due to the selection of ESBL-producing strains in our study, suggesting that these strains were more likely to carry fosfomycin resistance genes. Some studies indicated that the carrying of *fosA7* and *fosA3* was associated with specific *Salmonella* serotypes. *fosA7* was frequently found in *S. Agona*, *S. Derby*, and *S. Meleagridis*, while *fosA3* is commonly associated with *S. Typhimurium* and *S. Indiana* (Fang et al., 2020; Wang et al., 2022). This observation aligns with the findings of the present study, although it is noteworthy that 15 *S. Kentucky* strains were also found to carry the *fosA7* and *fosA3* genes, indicating that *S. Kentucky* strains might also exhibit susceptibility to *fosA3*. In China, florfenicol is a veterinary-specific antimicrobial drug, and Zhan et al. (2019) demonstrated the number of florfenicol-resistant strains had increased year by year. In this study, we also observed a high prevalence of the florfenicol-resistant gene *floR*, detected at rates of 100.00% in swine sources and 80.16% in broiler sources. This high prevalence might be attributed to the gene’s presence on multiple transposons (IS1006, ISEc59, ISVsa3, ISVsa, and IS5075).

## 5 Conclusion

Significant differences were observed in the dominant strains of *Salmonella* from swines and broilers prevalent in China from 2014 to 2023. Clonal transmission of these strains occurred across different years and provinces. The application of cgMLST typing technology proved to be valuable in tracking strains accurately. Additionally, the phenomenon of multi-drug resistance has increased, with broiler-origin *Salmonella* exhibiting a more pronounced resistance compared to swine-origin strains. It is worth noting that strains producing ESBLs have a high detection rate in *Salmonella* from broilers. Three serotypes (*S. Typhimurium*, *S. Indiana*, and *S. Kentucky*) were found to be more likely to produce ultra-broad-spectrum β-lactamases. This suggests that we should thoroughly implement management policies aimed at reducing the use of veterinary antimicrobials. Additionally, we should enhance research on traceability technology and the abatement of resistance genes, thereby providing support for the effective prevention and control of the spread of *Salmonella* and its drug resistance.

## Data Availability

The authors acknowledge that the data presented in this study must be deposited and made publicly available in an acceptable repository, prior to publication. Frontiers cannot accept a manuscript that does not adhere to our open data policies.

## Footnotes

1
http://bacdb.cn/bacwgstdb/tools.php

2
https://www.genomicepidemiology.org/

## References

[B1] Aslam B. Khurshid M. Arshad M. I. Muzammil S. Rasool M. Yasmeen N. (2021). Antibiotic resistance: One health one world outlook. *Front. Cell. Infect. Microbiol.* 11:771510. 10.3389/fcimb.2021.771510 34900756 PMC8656695

[B2] Clinical Laboratory Standards Institute (CLSI) (2020). *Performance standards for antimicrobial susceptibility testing*. CLSI document M100- Ed30. Malvern, PA: Clinical and Laboratory Standards Institute.

[B3] Deng L. Lv L.-C. Tu J. Yue C. Bai Y. He X. (2024). Clonal spread of *bla* NDM-1-carrying *Salmonella enterica* serovar Typhimurium clone ST34 and wide spread of IncHI2/ST3- *bla* NDM-5 plasmid in China. *J. Antimicrobial Chemother.* 79 1900–1909. 10.1093/jac/dkae178 38943539

[B4] Doosti A. Zohoor A. Chehelgerdi M. Mokhtari-Farsani A. (2016). Distribution of TEM-1 gene in *Salmonella enterica* Isolated from poultry carcasses in Iran. *Thai J. Vet. Med.* 46 9–15. 10.56808/2985-1130.2723

[B5] Elnekave E. Hong S. L. Lim S. Johnson T. J. Perez A. Alvarez J. (2020). Comparing serotyping with whole-genome sequencing for subtyping of non-typhoidal *Salmonella enterica*: A large-scale analysis of 37 serotypes with a public health impact in the USA. *Microb. Genomics* 6:mgen000425. 10.1099/mgen.0.000425 32845830 PMC7643971

[B6] Elumalai S. Muthu G. Selvam R. E. M. Ramesh S. (2014). Detection of TEM-, SHV- and CTX-M-type β-lactamase production among clinical isolates of *Salmonella* species. *J. Med. Microbiol.* 63 962–967. 10.1099/jmm.0.068486-0 24866367

[B7] Fang L.-X. Jiang Q. Deng G.-H. He B. Sun R.-Y. Zhang J.-F. (2020). Diverse and flexible transmission of *fosA3* associated with heterogeneous multidrug resistance regions in *Salmonella enterica* Serovar Typhimurium and Indiana isolates. *Antimicrob. Agents Chemother.* 64:e02001-19. 10.1128/AAC.02001-19 31712202 PMC6985750

[B8] Farhat M. Khayi S. Berrada J. Mouahid M. Ameur N. El-Adawy H. (2023). Salmonella enterica serovar gallinarum biovars pullorum and gallinarum in poultry: Review of pathogenesis, antibiotic resistance, diagnosis and control in the genomic era. *Antibiotics* 13:23. 10.3390/antibiotics13010023 38247582 PMC10812584

[B9] Ferrari R. G. Rosario D. K. A. Cunha-Neto A. Mano S. B. Figueiredo E. E. S. Conte-Junior C. A. (2019). Worldwide epidemiology of salmonella serovars in animal-based foods: A meta-analysis. *Appl. Environ. Microbiol*. 85, e00591–19. 10.1128/AEM.00591-19 31053586 PMC6606869

[B10] Franceschine L. Braga P. Monteiro G. Fonseca B. (2023). Molecular and phenotypic detection of the resistance profile to β-lactams and colistin of *Salmonella* spp. isolated from broilers’ litter. *Braz. J. Poult. Sci*. 25, 001–006. 10.1590/1806-9061-2022-1676

[B11] Gu D. Wang Z. Tian Y. Kang X. Meng C. Chen X. (2020). Prevalence of *Salmonella* isolates and their distribution based on whole-genome sequence in a chicken slaughterhouse in Jiangsu, China. *Front. Vet. Sci.* 7:29. 10.3389/fvets.2020.00029 32154275 PMC7046563

[B12] Hendriksen R. S. Vieira A. R. Karlsmose S. Lo, Fo Wong D. M. A. Jensen A. B. (2011). Global monitoring of *Salmonella* serovar distribution from the world health organization global foodborne infections network country data bank: Results of quality assured laboratories from 2001 to 2007. *Foodborne Pathogens Dis.* 8 887–900. 10.1089/fpd.2010.0787 21492021

[B13] Inns T. Ashton P. M. Herrera-Leon S. Lighthill J. Foulkes S. Jombart T. (2017). Prospective use of whole genome sequencing (WGS) detected a multi-country outbreak of *Salmonella* Enteritidis. *Epidemiol. Infect.* 145 289–298. 10.1017/S0950268816001941 27780484 PMC9507544

[B14] Lee W. Greig D. Day M. Chattaway M. Nair S. (2019). Characterisation of extended spectrum beta-lactamases (ESBL) resistance in multi-drug resistant Salmonella concord. *Access Microbiol*. 1:1A. 10.1099/acmi.ac2019.po0081

[B15] Li X. Jiang Y. Wu K. Zhou Y. Liu R. Cao Y. (2017). Whole-genome sequencing identification of a multidrug-resistant *Salmonella enterica* serovar Typhimurium strain carrying bla NDM-5 from Guangdong, China. *Infect. Genet. Evol.* 55 195–198. 10.1016/j.meegid.2017.09.005 28893688

[B16] Mulvey M. R. Soule G. Boyd D. Demczuk W. Ahmed R. (2003). Characterization of the first extended-spectrum beta-lactamase-producing *Salmonella* isolate identified in Canada. *J. Clin. Microbiol.* 41 460–462. 10.1128/JCM.41.1.460-462.2003 12517894 PMC149628

[B17] Murray C. J. L. Ikuta K. S. Sharara F. Swetschinski L. Robles Aguilar G. Gray A. (2022). Global burden of bacterial antimicrobial resistance in 2019: A systematic analysis. *Lancet* 399 629–655. 10.1016/S0140-6736(21)02724-0 35065702 PMC8841637

[B18] Quick J. Ashton P. Calus S. Chatt C. Gossain S. Hawker J. (2015). Rapid draft sequencing and real-time nanopore sequencing in a hospital outbreak of *Salmonella*. *Genome Biol.* 16:114. 10.1186/s13059-015-0677-2 26025440 PMC4702336

[B19] Rathnayaka R. (2011). Evaluation of five DNA extraction methods in the detection of *Salmonella enterica* from meat using nested PCR. *J. Agric. Sci.* 6 24–31. 10.4038/jas.v6i1.3809

[B20] Schürch A. C. Arredondo-Alonso S. Willems R. J. L. Goering R. V. (2018). Whole genome sequencing options for bacterial strain typing and epidemiologic analysis based on single nucleotide polymorphism versus gene-by-gene–based approaches. *Clin. Microbiol. Infect.* 24 350–354. 10.1016/j.cmi.2017.12.016 29309930

[B21] Tang K. W. K. Millar B. C. Moore J. E. (2023). Antimicrobial Resistance (AMR). *Br. J. Biomed. Sci.* 80:11387. 10.3389/bjbs.2023.11387 37448857 PMC10336207

[B22] Van Boeckel T. P. Pires J. Silvester R. Zhao C. Song J. Criscuolo N. G. (2019). Global trends in antimicrobial resistance in animals in low- and middle-income countries. *Science* 365:eaaw1944. 10.1126/science.aaw1944 31604207

[B23] Wang D. Fang L.-X. Jiang Y.-W. Wu D.-S. Jiang Q. Sun R.-Y. (2022). Comparison of the prevalence and molecular characteristics of *fosA3* and *fosA7* among *Salmonella* isolates from food animals in China. *J. Antimicrob. Chemother.* 77 1286–1295. 10.1093/jac/dkac061 35296898

[B24] Wang Z. He J. Li Q. Tang Y. Wang J. Pan Z. (2020). First Detection of NDM-5-positive *Salmonella enterica* serovar typhimurium isolated from retail Pork in China. *Microbial Drug Resistance* 26 434–437. 10.1089/mdr.2019.0323 31682175

[B25] Whiley H. Ross K. (2015). *Salmonella* and eggs: From production to plate. *IJERPH* 12 2543–2556. 10.3390/ijerph120302543 25730295 PMC4377917

[B26] Wu H. Wang Y. Wu Y. Qiao J. Li H. Zheng S. (2015). Emergence of β-Lactamases and extended-spectrum β-Lactamases (ESBLs) producing *Salmonella* in retail raw chicken in China. *Foodborne Pathogens Dis.* 12 228–234. 10.1089/fpd.2014.1859 25658910

[B27] Yan S. Zhang W. Li C. Liu X. Zhu L. Chen L. (2021). Serotyping, MLST, and core genome MLST analysis of *Salmonella enterica* from different sources in China during 2004–2019. *Front. Microbiol.* 12:688614. 10.3389/fmicb.2021.688614 34603224 PMC8481815

[B28] Yang B. Wang Q. Cui S. Wang Y. Shi C. Xia X. (2014). Characterization of extended-spectrum beta-lactamases-producing *Salmonella* strains isolated from retail foods in Shaanxi and Henan Province, China. *Food Microbiol.* 42 14–18. 10.1016/j.fm.2014.02.003 24929711

[B29] Zhan Z. Xu X. Shen H. Gao Y. Zeng F. Qu X. (2019). Rapid emergence of florfenicol-resistant invasive non-typhoidal *Salmonella* in China: A potential threat to public health. *Am. J. Trop. Med. Hygiene* 101 1282–1285. 10.4269/ajtmh.19-0403 31642424 PMC6896861

[B30] Zhang P. Shen Z. Zhang C. Song L. Wang B. Shang J. (2017). Surveillance of antimicrobial resistance among *Escherichia coli* from chicken and swine, China, 2008–2015. *Vet. Microbiol.* 203 49–55. 10.1016/j.vetmic.2017.02.008 28619166

[B31] Zhang Y. Zhang N. Wang M. Luo M. Peng Y. Li Z. (2023). The prevalence and distribution of aminoglycoside resistance genes. *Biosaf. Health* 5 14–20. 10.1016/j.bsheal.2023.01.001 PMC11895043 40078603

[B32] Zhao Q. Sun H. Chen C. (2024). Current status and research strategies on the national campaign for reducing the use of veterinary antimicrobials for laying hens in China. *Chin. J. Veterinary Drug* 58 81–88. 10.11751/ISSN.1002-1280.2021.02.12

[B33] Zhao Y. Li Y. Song C. (2018). Establishment and application of PCR assay for serotype identification of *Salmonella*. *China Anim. Health Inspect.* 35 73–77. 10.3969/j.issn.1005-944X.2018.01.021

